# Outdoor artificial light at night exposure and gestational diabetes mellitus: a case–control study

**DOI:** 10.3389/fpubh.2024.1396198

**Published:** 2024-04-10

**Authors:** Qi Sun, Fang Ye, Jing Liu, Yang Yang, Qin Hui, Yuanmei Chen, Die Liu, Jianning Guo, Chao Wang, Di Lv, Lijuan Tang, Qi Zhang

**Affiliations:** ^1^National Center for Respiratory Medicine, State Key Laboratory of Respiratory Health and Multimorbidity, Department of Pediatrics, China-Japan Friendship Hospital, National Clinical Research Center for Respiratory Diseases, Institute of Respiratory Medicine, Chinese Academy of Medical Sciences, Beijing, China; ^2^Precision and Smart Imaging Laboratory, Beijing Friendship Hospital, Capital Medical University, Beijing, China; ^3^Graduate School of Peking Union Medical College, Chinese Academy of Medical Sciences, Beijing, China

**Keywords:** gestational diabetes mellitus, outdoor artificial light, pregnancy, risk factors, air pollution

## Abstract

**Objective:**

This study aims to explore the association between outdoor artificial light at night (ALAN) exposure and gestational diabetes mellitus (GDM).

**Methods:**

This study is a retrospective case–control study. According with quantiles, ALAN has been classified into three categories (Q1-Q3). GDM was diagnosed through oral glucose tolerance tests. Conditional logistic regression models were used to evaluate the association between ALAN exposure and GDM risk. The odds ratio (OR) with 95% confidence interval (CI) was used to assess the association. Restricted cubic spline analysis (RCS) was utilized to investigate the no liner association between ALAN and GDM.

**Results:**

A total of 5,720 participants were included, comprising 1,430 individuals with GDM and 4,290 matched controls. Pregnant women exposed to higher levels of ALAN during the first trimester exhibited an elevated risk of GDM compared to those with lower exposure levels (Q2 OR = 1.39, 95% CI 1.20–1.63, *p* < 0.001); (Q3 OR = 1.70, 95% CI 1.44–2.00, *p* < 0.001). Similarly, elevated ALAN exposure during the second trimester also conferred an increased risk of GDM (second trimester: Q2 OR = 1.70, 95% CI 1.45–1.98, *p* < 0.001; Q3 OR = 2.08, 95% CI 1.77–2.44, *p* < 0.001). RCS showed a nonlinear association between ALAN exposure and GDM risk in second trimester pregnancy, with a threshold value of 4.235.

**Conclusion:**

Outdoor ALAN exposure during pregnancy is associated with an increased risk of GDM.

## 1 Introduction

Exposure to artificial light at night (ALAN) has emerged as a progressively ubiquitous environmental hazard within contemporary society (1). Over the past several decades, urbanization and shifts in modern lifestyle have led to a continuous escalation of ALAN in our daily lives (2). While ALAN offers convenience and safety, it also brings forth an array of potential health concerns (3).

It is worth noting that recent research has employed satellite remote sensing data to validate the correlations between ALAN and a range of human health issues, including obesity (4), metabolic syndrome (5), sleep disorder (6, 7), and cancer (8). Furthermore, emerging evidence suggests an association between ALAN and the risk of type 2 diabetes (Minjee (9–11)). However, the relationship between outdoor ALAN exposure and gestational diabetes mellitus (GDM) remains poorly understood.

The mechanisms through which ALAN impacts human health remain unclear; however, research indicates that ALAN can disrupt circadian rhythms in humans and other organisms, thereby influencing various physiological processes and behavioral patterns (12, 13). Exposure to ALAN may even lead to suppressed secretion of melatonin, a hormone that plays a crucial role in regulating sleep and other physiological functions (14). Furthermore, ALAN may impact the functioning of other endocrine systems, such as the secretion of adrenal corticosteroids and insulin regulation (15).

GDM is a condition characterized by abnormal blood glucose levels during pregnancy (16). Reports indicate that the prevalence of GDM varies across different countries and regions, with a notably higher incidence of 14.8% reported in China, making it a noteworthy public health concern in the country (17). This increased prevalence can primarily be attributed to behavioral and environmental risk factors (18). For mothers, having GDM can lead to heightened risks of pregnancy complications such as hypertension (19) and preterm birth (20), along with an elevated risk of developing type 2 diabetes later in life (21). Additionally, GDM can have enduring consequences for the newborn, including neonatal cardiovascular health (22) and respiratory distress syndrome (23). Consequently, the identification of potential risk factors for gestational diabetes is of paramount importance in mitigating the risks posed to both mothers and their offspring.

Pregnant women constitute a unique population group, as they are more susceptible to the influence of environmental factors during pregnancy due to hormonal effects (24). Current research suggests that exposure to ALAN may have adverse effects on fetal size and the metabolism of offspring (25, 26). Hence, this study postulates that ALAN among pregnant women may is the risk of GDM through alterations in circadian rhythms and metabolism. The primary objective of this study is to investigate the association between outdoor ALAN exposure and gestational diabetes, aiming to address existing knowledge gaps and offer pertinent public health recommendations.

## 2 Materials and methods

### 2.1 Study population

**Figure 1:**
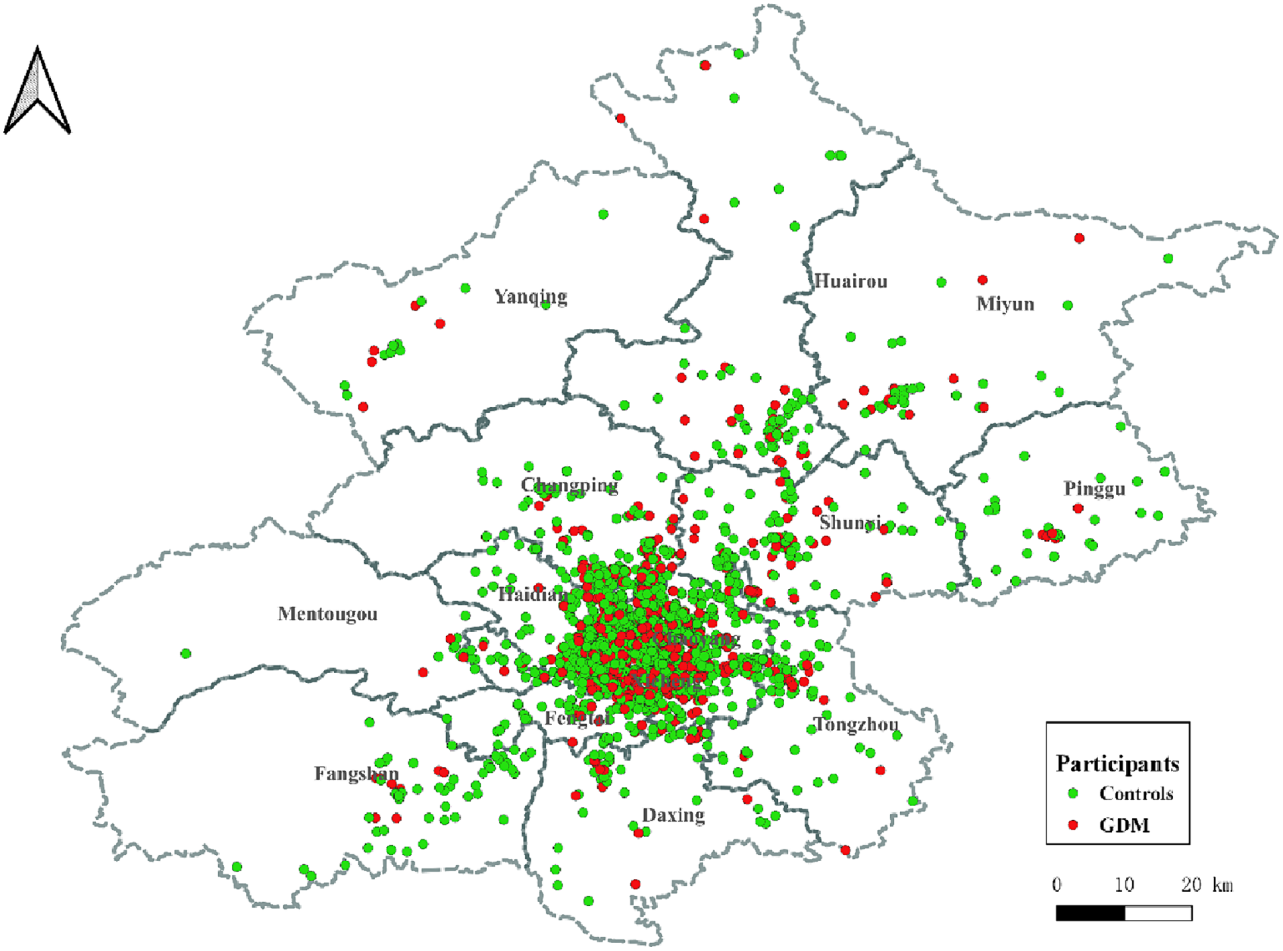
Geographical distribution of participants in Beijing. ALAN: artificial light at night; Red dots represent GDMs, and green dots represent controls. GDM, gestational diabetes mellitus.

**Figure 2:**
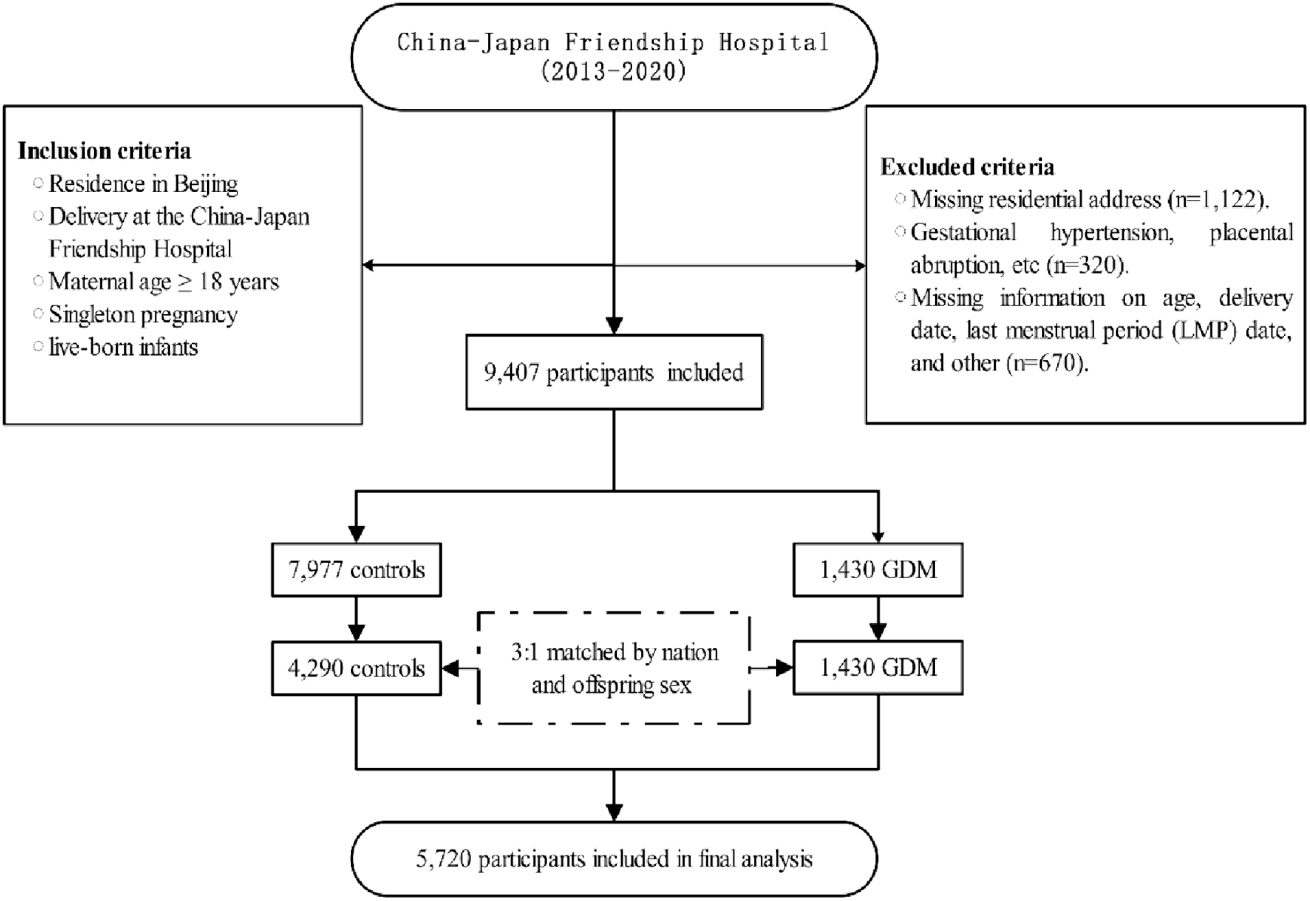
Flowchart of the study. LMP, Last Menstrual Period; GDM, Gestational diabetes mellitus; NDVI, normalized difference vegetation index; PM_2.5_, ambient fine particulate matter; PM_10_, ambient inhalable particulate matter.

This retrospective case–control study was conducted at the China-Japan Friendship Hospital. The geographic distribution of the study participants is illustrated in Figure 1. Participants were selected based on specific inclusion criteria, which included: (1) residence in Beijing; (2) delivery at the China-Japan Friendship Hospital; (3) maternal age ≥ 18 years; (4) singleton pregnancies; (5) live-born infants. Exclusion criteria encompassed: (1) missing residential address (*n* = 1,122); (2) presence of complications during pregnancy, such as gestational hypertension, placental abruption, etc. (*n* = 320); (3) missing information on age, delivery date, last menstrual period (LMP) date, and other related data (*n* = 670). A 1:3 propensity score matching was performed based on nation and offspring sex to select the control group. The final study comprised 5,720 participants, and the workflow is depicted in Figure 2.

The retrospective case–control study design precluded the acquisition of informed consent from the participants. Nevertheless, this approach aligns with the ethical review approved by the Ethics Committee of the China-Japan Friendship Hospital (Ethics Review Number: 2023-KY-137), which acknowledges the impracticality of obtaining informed consent in retrospective research studies.

### 2.2 Assessment of outdoor ALAN

In this study, ALAN measurements were obtained using the Suomi National Polar-Orbiting Partnership Visible Infrared Imaging Radiometer Suite (NPP-VIIRS), which offers superior spatial resolution, enhanced temporal resolution, an extended spectral range, and advanced calibration and correction when compared to the Operational Linescan System of Defense Meteorological Satellite Program (OLS-DMSP) (27). Commencing in April 2012, NPP-VIIRS captures data within the wavelength range of 500 nm to 900 nm, with a spatial resolution of 500 m × 500 m at the Equator (28). Monthly NPP-VIIRS nighttime light data for the period from 2013 to 2020 were obtained from the Earth Observation Group.^1^ The unit of measurement is nanowatts per square centimeter per steradian (nW/cm^2^/sr), which quantifies the radiative intensity per unit area, accounting for solid angles in all directions.

### 2.3 Outcomes and covariates

In this study, we directly acquired the diagnosis of GDM in participants from electronic health records. This diagnosis was based on the results of the 75 g oral glucose tolerance test (75 g OGTT) conducted on participants between gestational weeks 24–28. Participants were diagnosed with GDM if they met any of the following diagnostic criteria: fasting blood glucose level ≥ 5.1 mmol/L (92 mg/dL); 1-h blood glucose level ≥ 10.0 mmol/L (180 mg/dL); 2-h blood glucose level ≥ 8.5 mmol/L (153 mg/dL) (29). This study concurrently collected data on fetal sex and birth weight. Additionally, information on the following covariates was gathered: maternal race (Han, non-Han), age (years), parity (primiparous, multiparous), gravidity (1, 2, >2 times), pre-pregnancy body mass index (BMI, kg/m^2^), and conception season (Spring, Summer, Autumn, and Winter).

### 2.4 Other environmental variables

Given the role of environmental factors in GDM, we incorporated environmental covariates including inhalable particulate matter (PM_10_) and fine particulate matter (PM_2.5_), as well as green space, into the study. The data for PM_2.5_ and PM_10_ were sourced from the China High-resolution Air Pollutants (CHAP) database. PM_2.5_ and PM_10_ data were obtained using a spatiotemporal extreme random tree model that leveraged model data to fill spatial gaps in Moderate Resolution Imaging Spectroradiometer Multi-Angle Implementation of Atmospheric Correction Aerosol Optical Depth satellite products. This approach integrated ground observations, atmospheric reanalysis, emissions inventories, and other large-scale data sources, generating seamless nationwide surface PM_2.5_ and PM_10_ data from 2000 to 2021. The ten-fold cross-validation coefficient of determination (R^2^) for PM_2.5_ data was 0.92, with a root mean square error (RMSE) of 10.76 μg/m^3^ (30). For the PM_10_ data, the ten-fold cross-validation yielded an R^2^ of 0.9 and an RMSE of 21.12 μg/m^3^ (31). The Normalized Difference Vegetation Index (NDVI) was employed as a surrogate indicator for residential greenness. NDVI is a widely utilized metric in environmental research for quantifying the density and health status of vegetation in various regions (32). This index ranges from 0 to 1, where higher NDVI values indicate denser and healthier vegetation, while lower values suggest sparse or stressed vegetation (33). In our study, NDVI was estimated based on 16-day composite images from the NASA Terra Moderate Resolution Imaging Spectroradiometer satellite.^2^ After obtaining annual data for PM_2.5_, PM_10,_ and NDVI, we performed weighting matching for the residential locations of pregnant women and computed annual prenatal environmental pollution exposures.

### 2.5 Exposure time window

Participants’ residential addresses were geocoded using Baidu Maps.^3^ Subsequently, we proceeded to estimate the average exposure levels during the first and second trimesters of pregnancy to investigate potential heterogeneity in the association between ALAN and GDM across different exposure windows. These exposure windows corresponded to the first and second trimesters of pregnancy, corresponding to 3 and 6 months after the last menstrual period, respectively.

### 2.6 Statistical analysis

Continuous variables, normally distributed, are presented as mean ± standard deviation, while categorical variables are presented as counts (percentages). Differences between groups for continuous variables were compared using t-tests or Wilcoxon tests. Differences between groups for categorical variables were compared using chi-square tests or Fisher’s exact tests.

We employed conditional logistic regression to assess the link between ALAN exposure and GDM, calculating odds ratios (ORs) with 95% confidence intervals (CIs). Initially, we established an unadjusted model, without considering any potential confounding factors. Subsequently, we adjusted for potential confounders including age, ethnicity, gravidity, parity, pre-pregnancy body mass index, and conception season. Covariate selection guided by Directed Acyclic Graph Analysis (Supplementary Figure S1). Finally, while controlling for potential confounding, we further controlled for PM_2.5_, PM_10_, and NDVI. Employing Pearson correlation analysis, we identified a strong correlation between PM_2.5_ and PM_10_ (correlation coefficient = 0.97, *p* < 0.001). To mitigate issues of multicollinearity, principal component analysis was utilized to reduce the dimensionality of PM_2.5_ and PM_10_, incorporating the first principal component (PC1), which accounted for 71.65% of the variance, into the final model as a substitute for both PM_10_ and PM_2.5_.

To investigate the association between exposure to ALAN and GDM, restricted cubic spline (RCS) analysis was utilized in this study. The analysis was focused on ALAN exposure in first and second trimester pregnancy, assessing its nonlinear relationship with the risk of GDM. Additionally, we conducted a stratified analysis by infant sex to examine potential effect modification and assessed the interaction between ALAN and infant sex. The inclusion of interaction terms in the model was employed to assess whether fetal sex modifies the effect of exposure on the risk of GDM.

All statistical analyses were performed using R (version 4.1.0, available at https://www.r-project.org/).

### 2.7 Sensitivity analyses

This study conducted multiple sensitivity analyses: (1) ALAN per SD increase was employed to assess the relationship with GDM (Supplementary Tables S1, S2). (2) Evaluation of Han ethnicity participants was performed to assess potential influences related to ethnicity (Supplementary Table S3). (3) Similar analyses were conducted within the primiparous population to assess potential differences that might arise from multiple pregnancies (Supplementary Table S4). (4) Excluding participants with pre-existing diabetes prior to pregnancy (Supplementary Table S5). (5) Using linear regression to investigate the effect of ALAN exposure on participants’ fasting blood glucose levels (Supplementary Table S6).

## 3 Results

### 3.1 Characteristics of the study population

Table 1 provides an overview of the characteristics of pregnant women and newborns in the control group (*n* = 4,290) and GDM group (*n* = 1,430). While there were no significant differences in Han Chinese ethnicity between the group, the GDM group had a slightly higher mean age (GDM: 31.85 ± 3.96 years; Controls: 30.69 ± 3.41 years, *p* < 0.001). Furthermore, the GDM group showed a higher proportion of multiparous women (23.92% compared to 19.91% in the control group, *p* = 0.001). Gravidity distribution also significantly differed between the groups (*p* < 0.001). The distribution of neonatal sex was similar, with 51.40% males in the control group and 51.89% males in the GDM group. Additionally, there were slight differences in neonatal length (Control: 50.67 ± 2.39 cm; GDM: 50.47 ± 2.51 cm, *p* = 0.007), birth weight (Control: 3302.70 ± 479.89 g; GDM: 3270.36 ± 510.18 g, *p* = 0.030), and gestation duration (Control: 276.77 ± 12.90 days; GDM: 274.92 ± 33.59 days, *p* = 0.003) between the groups.

**Table 1:** Characteristics of pregnant women and newborns.

Variables		Controls (*n* = 4,290)	GDM (*n* = 1,430)	*p*
Han Chinese	No	208 (4.85)	79 (5.52)	0.345
	Yes	4,082 (95.15)	1,351 (94.48)	
Age (years)		30.69 ± 3.41	31.85 ± 3.96	< 0.001
Multipara	No	3,436 (80.09)	1,088 (76.08)	0.001
	Yes	854 (19.91)	342 (23.92)	
Gravidity (times)	1	2,702 (62.98)	817 (57.13)	< 0.001
	2	1,002 (23.36)	372 (26.01)	
	>2	586 (13.66)	241 (16.85)	
FBG (mmol/L)		4.51 ± 0.50	5.55 ± 0.79	< 0.001
BMI (kg/m^2^)		20.74 ± 2.65	21.31 ± 3.02	< 0.001
Neonatal sex	Male	2,205 (51.40)	742 (51.89)	0.772
	Female	2085 (48.60)	688 (48.11)	
Neonatal length (cm)		50.67 ± 2.39	50.47 ± 2.51	0.007
Birth weight (g)		3302.70 ± 479.89	3270.36 ± 510.18	0.030
Gestation (days)		276.77 ± 12.90	274.92 ± 33.59	0.003
Conception Season (%)	Spring	1,082 (25.22)	383 (26.78)	0.045
	Summer	1,149 (26.78)	345 (24.13)	
	Autumn	904 (21.07)	338 (23.64)	
	Winter	1,155 (26.92)	364 (25.45)	

Values are presented as mean ± standard deviation or count (percentage). GDM, gestational diabetes mellitus; FBG, Fasting Blood Glucose; BMI, Body Mass Index.

### 3.2 Distribution of environmental factors in different trimesters

Table 2 presents the differences in outdoor ALAN levels between the GDM and Control groups. There were no statistically significant differences in PM_10_ levels (Control: 102.85 ± 21.33 μg/m^3^; Case: 103.41 ± 20.70 μg/m^3^, *p* = 0.391) or PM_2.5_ levels (Control: 64.87 ± 17.72 μg/m^3^; Case: 65.90 ± 17.47 μg/m^3^, *p* = 0.054) between the two groups. Similarly, the NDVI showed no significant difference (Control: 0.32 ± 0.07; Case: 0.31 ± 0.07, *p* = 0.216). However, there were substantial differences in ALAN levels between the groups. In the first trimester (T1), ALAN levels were significantly higher in the GDM group (27.46 ± 16.86 nW/cm^2^/sr) compared to the Control group (24.42 ± 16.64 nW/cm^2^/sr, *p* < 0.001). This trend was consistent in the second trimester (T2) (Control: 24.69 ± 16.81 nW/cm^2^/sr; Case: 27.34 ± 16.61 nW/cm^2^/sr, *p* < 0.001).

**Table 2:** Differences in outdoor ALAN levels between the GDM and control groups.

Variables		Control group	Case group	*p*
PM_10_ (μg/m^3^)		102.85 ± 21.33	103.41 ± 20.70	0.391
PM_2.5_ (μg/m^3^)		64.87 ± 17.72	65.90 ± 17.47	0.054
NDVI		0.32 ± 0.07	0.31 ± 0.07	0.216
ALAN _T1_ (nW/cm^2^/sr)		24.42 ± 16.64	27.46 ± 16.86	< 0.001
ALAN _T2_ (nW/cm^2^/sr)		24.69 ± 16.81	27.34 ± 16.61	< 0.001
ALAN _T1_ category (%)	Q1	1,510 (35.20)	398 (27.83)	< 0.001
	Q2	1,414 (32.96)	491 (34.34)	
	Q3	1,366 (31.84)	541 (37.83)	
ALAN _T2_ category (%)	Q1	1,499 (34.94)	409 (28.60)	< 0.001
	Q2	1,432 (33.38)	473 (33.08)	
	Q3	1,359 (31.68)	548 (38.32)	

ALAN, Artificial Light at Night; NDVI, Normalized Difference Vegetation Index; T1, First Trimester; T2, Second; Q1-Q3, Categorized into three groups based on percentiles.

### 3.3 Association of outdoor ALAN exposure in different trimesters with GDM

**Figure 3:**
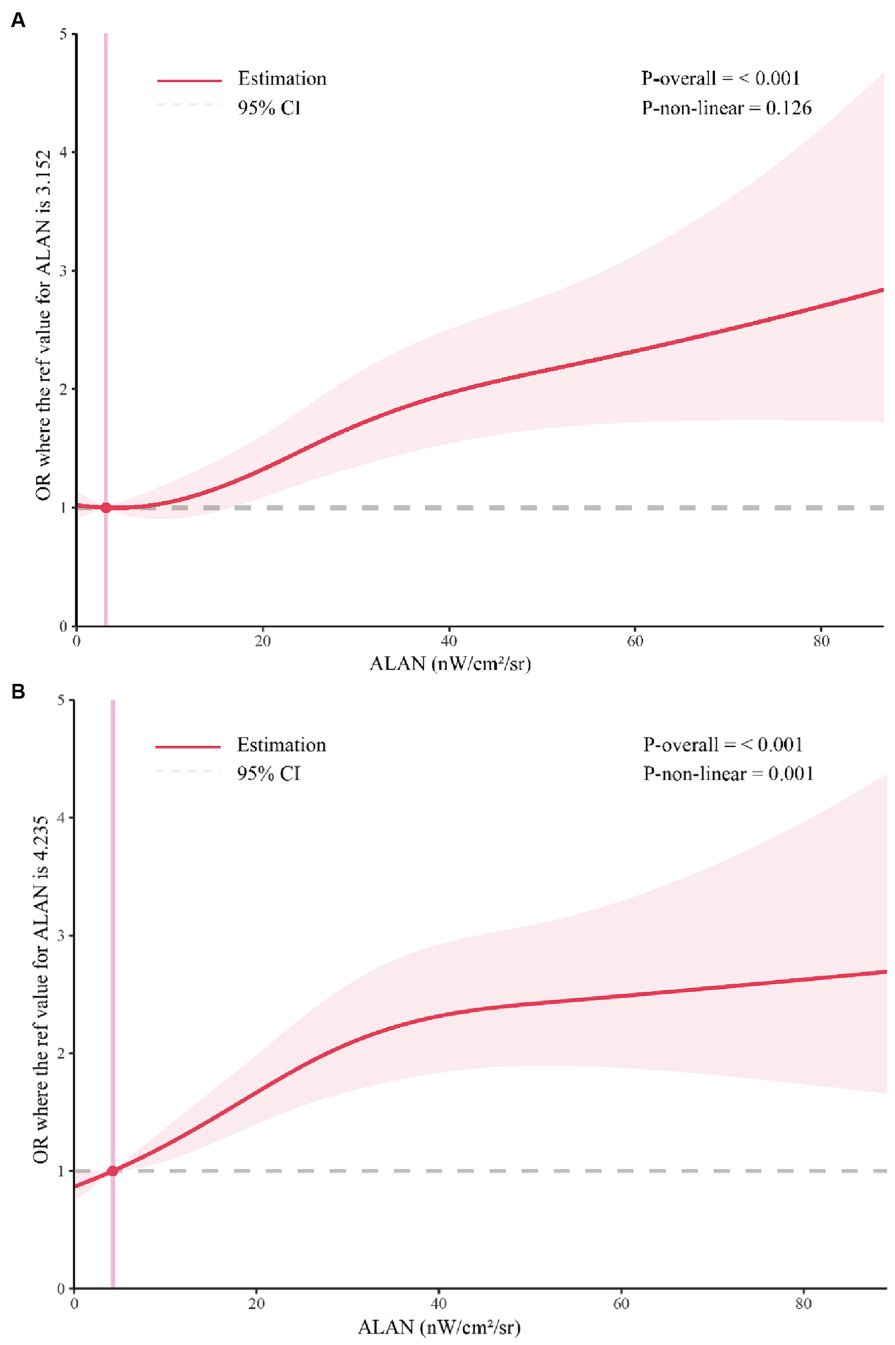
Restricted cubic spline analysis. **(A)** The association between first-trimester ALAN and GDM; **(B)** The relationship between second trimester ALAN and GDM; ALAN, Artificial Light at Night; GDM, Gestational Diabetes Mellitus.

In Table 3, we present the results of conditional logistic regression models examining the association between outdoor ALAN exposure and the risk of GDM across various trimesters (T1 and T2). In the initial unadjusted model (Model 1), participants in the second (Q2) and third (Q3) quartiles of ALAN exposure exhibited significantly elevated odds of developing GDM compared to those in the first quartile (Q1) during all trimesters (all *p*-values <0.001). These results remained consistent after accounting for potential confounders. Specifically, for the first trimester, the ORs were as follows: Q2 OR = 1.39 (95%CI 1.20–1.63, *p* < 0.001), Q3 OR = 1.70 (95%CI 1.44, 2.00, *p* < 0.001). In the second trimester, the ORs were: Q2 OR = 1.70 (95%CI 1.45–1.98, *p* < 0.001), Q3 OR = 2.08 (95%CI 1.77–2.44, *p* < 0.001). No significant interaction between ALAN exposure and sex was observed across all models. Table 4 presents the sex-specific associations of ALAN exposure with the risk of GDM across different trimesters, along with tests for interaction. ALAN exposure exhibited consistent associations with GDM risk across trimesters, particularly among females. In our study, RCS analysis showed no significant nonlinear relationship between ALAN exposure and GDM risk in first trimester pregnancy. However, a significant nonlinear association was found in second trimester pregnancy, with a threshold value of 4.235 (Figure 3).

**Table 3:** Association of outdoor ALAN exposure with GDM.

		OR (95%CI)	*p*	*P* for trend
Model 1				
ALAN _T1_	Q1	ref		<0.001
	Q2	1.33 (1.15, 1.55)	<0.001	
	Q3	1.54 (1.32, 1.80)	<0.001	
ALAN _T2_	Q1	ref		<0.001
	Q2	1.23 (1.05, 1.43)	0.009	
	Q3	1.51 (1.30, 1.76)	<0.001	
Model 2				
ALAN _T1_	Q1	ref		<0.001
	Q2	1.39 (1.20, 1.61)	<0.001	
	Q3	1.72 (1.47, 2.01)	<0.001	
ALAN _T2_	Q1	ref		<0.001
	Q2	1.57 (1.36, 1.82)	<0.001	
	Q3	1.89 (1.63, 2.20)	<0.001	
Model 3				
ALAN _T1_	Q1	ref		<0.001
	Q2	1.39 (1.20, 1.63)	<0.001	
	Q3	1.70 (1.44, 2.00)	<0.001	
ALAN _T2_	Q1	ref		<0.001
	Q2	1.70 (1.45, 1.98)	<0.001	
	Q3	2.08 (1.77, 2.44)	<0.001	

GDM, gestational diabetes mellitus; ALAN, artificial light at night; T1, First Trimester; T2, Second Trimester; Q1-Q3, Categorized into three groups based on percentiles; OR, Odds ratio; 95%CI, 95% confidence interval. Model 1: Crude conditional logistic regression model; Model 2: Adjusted for age, ethnicity, gravidity, parity, pre-pregnancy body mass index, and conception season; Model 3: Based on model 2, further adjusted for normalized difference vegetation index (NDVI), as well as Principal Component 1 of ambient fine particulate matter (PM2.5) and ambient inhalable particulate matter (PM10).

**Table 4:** Sex-specific associations of ALAN exposure with GDM.

		Male			Female			*P* for interaction
		OR (95%CI)	*p*	*P* for trend	OR (95%CI)	*p*	*P* for trend	
Model 1								
ALAN _T1_	Q1			0.100			<0.001	
	Q2	1.25 (0.98, 1.60)	0.077		1.73 (1.33, 2.27)	<0.001		0.137
	Q3	1.24 (0.96, 1.60)	0.094		1.86 (1.43, 2.43)	<0.001		0.135
ALAN _T2_	Q1			0.003			0.003	
	Q2	1.10 (0.86, 1.41)	0.455		1.11 (0.86, 1.44)	0.430		0.673
	Q3	1.46 (1.14, 1.87)	0.003		1.47 (1.13, 1.92)	0.004		0.883
Model 2				0.104			0.004	
ALAN _T1_	Q1	1.10 (0.79, 1.53)	0.583		2.08 (1.45, 2.98)	<0.001		0.153
	Q2	1.34 (0.92, 1.94)	0.122		1.86 (1.27, 2.72)	0.001		0.321
	Q3			0.001			0.003	
ALAN _T2_	Q1	1.27 (0.92, 1.74)	0.148		1.48 (1.04, 2.10)	0.028		0.548
	Q2	1.75 (1.24, 2.48)	0.001		1.79 (1.23, 2.60)	0.002		0.977
	Q3						<0.001	
Model 3		0.98 (0.74, 1.29)	0.887		2.51 (1.84, 3.42)	<0.001		0.101
ALAN _T1_	Q1	1.05 (0.79, 1.41)	0.730		2.17 (1.58, 2.98)	<0.001		0.127
	Q2			0.004			<0.001	
	Q3	1.22 (0.93, 1.61)	0.149		1.83 (1.35, 2.47)	<0.001		0.183
ALAN _T2_	Q1	1.65 (1.25, 2.19)	<0.001		2.44 (1.78, 3.36)	<0.001		0.515
	Q2			0.135			<0.001	
	Q3	0.72 (0.47, 1.00)	0.051		1.86 (1.37, 2.52)	<0.001		0.221

GDM, gestational diabetes mellitus; ALAN, artificial light at night; T1, First Trimester; T2, Second Trimester; Q1-Q3, Categorized into three groups based on percentiles; OR, Odds ratio; 95%CI, 95% confidence interval. Model 1: Crude conditional logistic regression model; Model 2: Adjusted for age, ethnicity, gravidity, parity, pre-pregnancy body mass index, and conception season; Model 3: Based on model 2, further adjusted for normalized difference vegetation index (NDVI), as well as Principal Component 1 of ambient fine particulate matter (PM2.5) and ambient inhalable particulate matter (PM10).

## 4 Discussion

To investigate the association between outdoor ALAN exposure and GDM, we conducted a retrospective case–control study. Our study found a significant association between exposure to outdoor ALAN during pregnancy and an increased risk of GDM after adjusting for confounding factors. Furthermore, the association between outdoor ALAN and the risk of GDM did not differ between male and female infants. Our findings provide evidence supporting the role of outdoor ALAN in the risk of GDM among pregnant women.

In recent decades, the impact of ALAN on human health has gained global attention. Numerous studies have investigated the associations between ALAN exposure and chronic conditions such as cardiovascular diseases (34), obesity (35), and mental disorders (36). Recent research has suggested that exposure to outdoor ALAN may increase the risk of type 2 diabetes mellitus (T2DM) (Minjee (9, 10)). Furthermore, a cross-sectional study has shown a significant association between long-term exposure to higher-intensity outdoor ALAN and an increased risk of impaired glucose metabolism (11). Recent studies have elucidated the relationship between ALAN and GDM. In the United States, the risk associated with GDM has been correlated with pre-sleep exposure to light, as measured by wrist-worn activity monitors (37). Consistent with our findings, a prospective cohort study in Sichuan Province, China, utilizing satellite data to estimate outdoor ALAN exposure, offered a broader perspective on environmental exposure (38). Furthermore, a study conducted in Hefei City revealed that outdoor ALAN was associated with elevated early-pregnancy glucose homeostasis markers, yet it did not correlate with GDM risk (39). The variability in these findings may be attributed to differences in study populations and geographical locations. Our research, conducted in Beijing, a major metropolitan area, underscores the significant public health implications of addressing light pollution in densely populated urban environments. Moreover, our study surpassed traditional methods by thoroughly adjusting for critical environmental variables, including PM_2.5_, PM_10_, and NDVI, thereby reinforcing the robustness and credibility of our findings.

Exploring the critical windows of association between maternal ALAN exposure and the risk of GDM is of paramount importance for devising targeted intervention measures. The early and mid-stages of pregnancy are crucial periods for embryonic and fetal development, being particularly susceptible to external environmental influences (40). In our study, we observed that pregnant women exposed to higher levels of ALAN during the first and second trimesters exhibited an increased risk of GDM. However, considering the timing of GDM diagnosis (41), the relationship between ALAN exposure during the second trimester of pregnancy and GDM may be subject to constraints, necessitating further investigation.

The mechanisms underlying the relationship between ALAN exposure during pregnancy and the risk of GDM remain poorly understood. Several potential mechanisms may be involved. Firstly, ALAN exposure could potentially impact the risk of GDM by disrupting the circadian rhythms of pregnant women. Circadian rhythm regulation during pregnancy is critical for normal fetal and maternal physiological processes (42). ALAN may induce circadian rhythm disruption (43), leading to sleep disturbances and reduced sleep quality among pregnant women, consequently increasing the risk of GDM. Secondly, hormonal changes may play a significant role. ALAN exposure may influence hormone levels in pregnant women (44), particularly melatonin, a hormone crucial for regulating circadian rhythms during pregnancy (45). ALAN exposure might suppress melatonin secretion, potentially affecting maternal physiology and fetal development negatively. Lastly, ALAN exposure may contribute to an elevated risk of GDM by provoking alterations in inflammation and immune responses. Animal experiments have demonstrated that prolonged illumination can lead to changes in both the immune system and inflammatory processes (46). Although these mechanisms remain multifaceted and not fully elucidated, further research is needed to unravel these intricate pathways. In-depth investigations in both laboratory and epidemiological settings will contribute to a better understanding of the relationship between ALAN exposure and GDM, offering more precise directions for future intervention strategies.

This study has several limitations that warrant discussion. Firstly, in our research, we estimated outdoor ALAN exposure during pregnancy using high-resolution satellite images. However, we lacked data on indoor light exposure and whether participants used blackout curtains during the night, which could potentially lead to exposure misclassification. Future studies should consider collecting information on both indoor and outdoor light exposure. Secondly, while we adjusted for environmental confounders related to GDM, such as environmental particulate matter (47) and greenness (48) at the residential area, we did not account for other potential confounding factors, such as temperature (49), household income and education level. The absence of this information needs to be addressed and improved in future research. Thirdly, our study adopted a retrospective case–control study design, limiting the ability to establish causality between ALAN exposure and GDM. Therefore, the relationship between ALAN and GDM needs further confirmation through prospective study designs. Fourthly, the annual inclusion of study participants was not uniform (Supplementary Table S7), which was due to the COVID-19 pandemic. Although the ratio of cases to controls remained consistent, this could potentially introduce a certain degree of bias. Finally, our single-center study involved participants from the Beijing area with relatively higher socioeconomic status. Caution is advised when extending the study results to regions with lower economic development. Future research should validate these findings in diverse socioeconomic contexts.

Despite these limitations, our study possesses several strengths. Firstly, we elucidated the association between ALAN exposure during pregnancy and GDM, identifying the critical exposure window for this relationship. This finding provides valuable reference for targeted intervention measures during the identified exposure window. Additionally, we conducted a series of sensitivity analyses and performed stratified analyses by newborn sex to assess the consistency and robustness of this relationship.

## 5 Conclusion

In summary, our study reveals that higher outdoor ALAN exposure during pregnancy is associated with an elevated risk of GDM. These findings emphasize the need for targeted interventions and further research to better understand the mechanisms underlying this relationship and mitigate the health risks associated with light pollution during pregnancy.

## Data availability statement

The raw data supporting the conclusions of this article will be made available by the authors, without undue reservation.

## Ethics statement

The studies involving humans were approved by Ethics Committee of the China-Japan Friendship Hospital. The studies were conducted in accordance with the local legislation and institutional requirements. The ethics committee/institutional review board waived the requirement of written informed consent for participation from the participants or the participants' legal guardians/next of kin because this was a retrospective study and the ethics committee waived informed consent.

## Author contributions

QS: Methodology, Writing – original draft, Writing – review & editing. FY: Investigation, Visualization, Writing – original draft, Writing – review & editing. JL: Investigation, Writing – original draft, Writing – review & editing. YY: Investigation, Writing – original draft, Writing – review & editing. QH: Software, Writing – original draft, Writing – review & editing YC: Data curation, Resources, Writing – original draft, Writing – review & editing. DLi: Software, Writing – original draft, Writing – review & editing. JG: Data Curation, Writing – original draft, Writing – review & editing. CW: Software, Writing – original draft, Writing – review & editing. DLv: Visualization, Writing – original draft, Writing – review & editing. LT: Investigation, Writing – original draft, Writing – review & editing. QZ: Conceptualization, Supervision, Writing – original draft, Writing – review & editing.

## Footnotes

^1^ https://eogdata.mines.edu/

^2^ https://ladsweb.modaps.eosdis.nasa.gov

^3^ https://map.baidu.com
