# Supplementary material for: Outdoor artificial light at night exposure and gestational diabetes mellitus: a case–control study
Source: Front Public Health. 2024 Apr 10;12:1396198. doi: 10.3389/fpubh.2024.1396198 (PMC11039930; doi:10.3389/fpubh.2024.1396198)
Supplement: Supplementary file 1 [file Data_Sheet_1.docx]

Supplementary Table S1. Association of outdoor ALAN per SD exposure with GDM

|  |  | OR (95%CI) | *P* |
| --- | --- | --- | --- |
| Model 1 |  |  |  |
| ALAN _T1_ | ALAN | 1.01 (1.01, 1.02) | <0.001 |
|  | ALAN per SD | 1.22 (1.14, 1.30) | <0.001 |
| ALAN _T2_ | ALAN | 1.01 (1.01, 1.01) | <0.001 |
|  | ALAN per SD | 1.17 (1.10, 1.25) | <0.001 |
| Model 2 |  |  |  |
| ALAN _T1_ | ALAN | 1.01 (1.01, 1.02) | <0.001 |
|  | ALAN per SD | 1.29 (1.19, 1.39) | <0.001 |
| ALAN _T2_ | ALAN | 1.01 (1.01, 1.02) | <0.001 |
|  | ALAN per SD | 1.28 (1.18, 1.38) | <0.001 |
| Model 3 |  |  |  |
| ALAN _T1_ | ALAN | 1.02 (1.01, 1.02) | <0.001 |
|  | ALAN per SD | 1.30 (1.20, 1.42) | <0.001 |
| ALAN _T2_ | ALAN | 1.02 (1.01, 1.02) | <0.001 |
|  | ALAN per SD | 1.33 (1.23, 1.44) | <0.001 |

GDM: gestational diabetes mellitus; ALAN: artificial light at night; T1: First Trimester; T2: Second Trimester; OR: Odds ratio; 95%CI: 95% confidence interval. Model 1: Crude conditional logistic regression model; Model 2: Adjusted for age, ethnicity, gravidity, parity, pre-pregnancy body mass index, and conception season; Model 3: Based on model 2, further adjusted for normalized difference vegetation index (NDVI), as well as Principal Component 1 of ambient fine particulate matter (PM_2.5_) and ambient inhalable particulate matter (PM_10_).

Supplementary Table S2. Sex-specific associations of ALAN per SD exposure with GDM

|  |  | Male | | Female | | *P* for interaction |
| --- | --- | --- | --- | --- | --- | --- |
|  |  | OR (95%CI) | *P* | OR (95%CI) | *P* |  |
| Model 1 |  |  |  |  |  |  |
| ALAN | T1 | 1.01 (1.00, 1.01) | 0.085 | 1.02 (1.01, 1.02) | <0.001 | 0.041 |
|  | T2 | 1.01 (1.00, 1.02) | 0.005 | 1.01 (1.00, 1.02) | 0.002 | 0.598 |
| ALAN per SD | T1 | 1.10 (0.99, 1.22) | 0.085 | 1.34 (1.20, 1.50) | <0.001 | 0.047 |
|  | T2 | 1.16 (1.05, 1.30) | 0.005 | 1.18 (1.06, 1.32) | 0.003 | 0.630 |
| Model 2 |  | 1.01 (1.00, 1.02) | 0.063 | 1.02 (1.01, 1.03) | <0.001 | 0.117 |
| ALAN | T1 | 1.01 (1.01, 1.02) | 0.001 | 1.01 (1.01, 1.02) | 0.002 | 0.738 |
|  | T2 | 1.01 (1.00, 1.01) | 0.170 | 1.02 (1.01, 1.03) | <0.001 | 0.038 |
| ALAN per SD | T1 | 1.26 (1.09, 1.46) | 0.002 | 1.27 (1.09, 1.49) | 0.003 | 0.769 |
|  | T2 | 1.14 (0.98, 1.32) | 0.088 | 1.36 (1.15, 1.60) | <0.001 | 0.031 |
| Model 3 |  | 1.01 (1.00, 1.02) | 0.004 | 1.02 (1.01, 1.03) | <0.001 | 0.069 |
| ALAN | T1 | 1.00 (0.99, 1.01) | 0.948 | 1.02 (1.01, 1.03) | <0.001 | 0.550 |
|  | T2 | 1.08 (0.93, 1.26) | 0.287 | 1.47 (1.24, 1.75) | <0.001 | 0.101 |
| ALAN per SD | T1 | 1.01 (0.87, 1.17) | 0.857 | 1.36 (1.15, 1.60) | <0.001 | 0.057 |
|  | T2 | 1.01 (1.00, 1.01) | 0.085 | 1.02 (1.01, 1.02) | <0.001 | 0.041 |

GDM: gestational diabetes mellitus; ALAN: artificial light at night; ALAN per SD: the effect of ALAN per standard deviation; T1: First Trimester; T2: Second Trimester; OR: Odds ratio; 95%CI: 95% confidence interval. Model 1: Crude conditional logistic regression model; Model 2: Adjusted for age, ethnicity, gravidity, parity, pre-pregnancy body mass index, and conception season; Model 3: Based on model 2, further adjusted for normalized difference vegetation index (NDVI), as well as Principal Component 1 of ambient fine particulate matter (PM_2.5_) and ambient inhalable particulate matter (PM_10_).

Supplementary Table S3. Association of outdoor ALAN exposure with GDM in Han-Chinese participants

|  |  | OR (95%CI) | *P* | *P* for trend |
| --- | --- | --- | --- | --- |
| Model 1 |  |  |  |  |
| ALAN _T1_ | Q1 | ref |  | <0.001 |
|  | Q2 | 1.33 (1.14, 1.56) | <0.001 |  |
|  | Q3 | 1.56 (1.33, 1.83) | <0.001 |  |
| ALAN _T2_ | Q1 | ref |  | <0.001 |
|  | Q2 | 1.23 (1.05, 1.44) | 0.011 |  |
|  | Q3 | 1.54 (1.31, 1.80) | <0.001 |  |
| Model 2 |  |  |  |  |
| ALAN _T1_ | Q1 | ref |  | <0.001 |
|  | Q2 | 1.36 (1.12, 1.66) | 0.002 |  |
|  | Q3 | 1.73 (1.40, 2.15) | <0.001 |  |
| ALAN _T2_ | Q1 | ref |  | <0.001 |
|  | Q2 | 1.53 (1.26, 1.86) | <0.001 |  |
|  | Q3 | 1.93 (1.56, 2.38) | <0.001 |  |
| Model 3 |  |  |  |  |
| ALAN _T1_ | Q1 | ref |  | <0.001 |
|  | Q2 | 1.35 (1.12, 1.62) | 0.001 |  |
|  | Q3 | 1.66 (1.36, 2.02) | <0.001 |  |
| ALAN _T2_ | Q1 | ref |  | <0.001 |
|  | Q2 | 1.40 (1.16, 1.68) | <0.001 |  |
|  | Q3 | 1.69 (1.39, 2.06) | <0.001 |  |

GDM: gestational diabetes mellitus; ALAN: artificial light at night; T1: First Trimester; T2: Second Trimester; Q1-Q3: Categorized into three groups based on percentiles; OR: Odds ratio; 95%CI: 95% confidence interval. Model 1: Crude conditional logistic regression model; Model 2: Adjusted for age, gravidity, parity, pre-pregnancy body mass index, and conception season; Model 3: Based on model 2, further adjusted for normalized difference vegetation index (NDVI), as well as Principal Component 1 of ambient fine particulate matter (PM_2.5_) and ambient inhalable particulate matter (PM_10_).

Supplementary Table S4. Association of outdoor ALAN exposure with GDM in primiparous participants

|  |  | OR (95%CI) | *P* | *P* for trend |
| --- | --- | --- | --- | --- |
| Model 1 |  |  |  |  |
| ALAN _T1_ | Q1 | ref |  | <0.001 |
|  | Q2 | 1.42 (1.18, 1.69) | <0.001 |  |
|  | Q3 | 1.50 (1.25, 1.80) | <0.001 |  |
| ALAN _T2_ | Q1 | ref |  | <0.001 |
|  | Q2 | 1.30 (1.09, 1.56) | 0.004 |  |
|  | Q3 | 1.45 (1.21, 1.74) | <0.001 |  |
| Model 2 |  |  |  |  |
| ALAN _T1_ | Q1 | ref |  | <0.001 |
|  | Q2 | 1.52 (1.29, 1.79) | <0.001 |  |
|  | Q3 | 1.68 (1.42, 2.00) | <0.001 |  |
| ALAN _T2_ | Q1 | ref |  | <0.001 |
|  | Q2 | 1.68 (1.42, 1.98) | <0.001 |  |
|  | Q3 | 1.82 (1.54, 2.15) | <0.001 |  |
| Model 3 |  |  |  |  |
| ALAN _T1_ | Q1 | ref |  | <0.001 |
|  | Q2 | 1.58 (1.33, 1.87) | <0.001 |  |
|  | Q3 | 1.69 (1.41, 2.02) | <0.001 |  |
| ALAN _T2_ | Q1 | ref |  | <0.001 |
|  | Q2 | 1.83 (1.54, 2.18) | <0.001 |  |
|  | Q3 | 2.02 (1.68, 2.41) | <0.001 |  |

GDM: gestational diabetes mellitus; ALAN: artificial light at night; T1: First Trimester; T2: Second Trimester; Q1-Q3: Categorized into three groups based on percentiles; OR: Odds ratio; 95%CI: 95% confidence interval. Model 1: Crude conditional logistic regression model; Model 2: Adjusted for age, ethnicity, gravidity, pre-pregnancy body mass index, and conception season; Model 3: Based on model 2, further adjusted for normalized difference vegetation index (NDVI), as well as Principal Component 1 of ambient fine particulate matter (PM_2.5_) and ambient inhalable particulate matter (PM_10_).

Supplementary Table S5. Association of outdoor ALAN exposure with GDM in participants without pre-pregnancy diabetes.

|  |  | OR (95%CI) | *P* | *P* for trend |
| --- | --- | --- | --- | --- |
| Model 1 |  |  |  |  |
| ALAN _T1_ | Q1 | ref |  | <0.001 |
|  | Q2 | 1.34 (1.15, 1.56) | <0.001 |  |
|  | Q3 | 1.54 (1.32, 1.80) | <0.001 |  |
| ALAN _T2_ | Q1 | ref |  | <0.001 |
|  | Q2 | 1.23 (1.06, 1.44) | 0.008 |  |
|  | Q3 | 1.51 (1.30, 1.76) | <0.001 |  |
| Model 2 |  |  |  |  |
| ALAN _T1_ | Q1 | ref |  | <0.001 |
|  | Q2 | 1.35 (1.11, 1.63) | 0.002 |  |
|  | Q3 | 1.66 (1.34, 2.05) | <0.001 |  |
| ALAN _T2_ | Q1 | ref |  | <0.001 |
|  | Q2 | 1.55 (1.28, 1.87) | <0.001 |  |
|  | Q3 | 1.84 (1.50, 2.25) | <0.001 |  |
| Model 3 |  |  |  |  |
| ALAN _T1_ | Q1 | ref |  | <0.001 |
|  | Q2 | 1.39 (1.13, 1.71) | 0.002 |  |
|  | Q3 | 1.72 (1.36, 2.19) | <0.001 |  |
| ALAN _T2_ | Q1 | ref |  | <0.001 |
|  | Q2 | 1.71 (1.39, 2.11) | <0.001 |  |
|  | Q3 | 2.10 (1.66, 2.65) | <0.001 |  |

GDM: gestational diabetes mellitus; ALAN: artificial light at night; T1: First Trimester; T2: Second Trimester T3: Third Trimester; Q1-Q3: Categorized into three groups based on percentiles; OR: Odds ratio; 95%CI: 95% confidence interval. Model 1: Crude conditional logistic regression model; Model 2: Adjusted for age, ethnicity, gravidity, parity, pre-pregnancy body mass index, and conception season; Model 3: Based on model 2, further adjusted for normalized difference vegetation index (NDVI), as well as Principal Component 1 of ambient fine particulate matter (PM_2.5_) and ambient inhalable particulate matter (PM_10_).

Supplementary Table S6. Association of outdoor ALAN exposure with fasting blood glucose using linear regression model

|  |  | β (95%CI) | *P* | *P* for trend |
| --- | --- | --- | --- | --- |
| Model 1 |  |  |  |  |
| ALAN _T1_ | Q1 | ref |  | <0.001 |
|  | Q2 | 0.047 (0.006, 0.087) | 0.011 |  |
|  | Q3 | 0.090 (0.043, 0.137) | <0.001 |  |
| ALAN _T2_ | Q1 | ref |  | 0.004 |
|  | Q2 | 0.047 (0.027, 0.067) | 0.001 |  |
|  | Q3 | 0.069 (0.023, 0.116) | 0.004 |  |
| Model 2 |  |  |  |  |
| ALAN _T1_ | Q1 | ref |  | 0.003 |
|  | Q2 | 0.054 (0.018, 0.090) | 0.024 |  |
|  | Q3 | 0.086 (0.028, 0.143) | <0.001 |  |
| ALAN _T2_ | Q1 | ref |  | 0.001 |
|  | Q2 | 0.052 (0.012, 0.092) | 0.013 |  |
|  | Q3 | 0.092 (0.036, 0.148) | 0.001 |  |
| Model 3 |  |  |  |  |
| ALAN _T1_ | Q1 | ref |  | <0.001 |
|  | Q2 | 0.058 (0.005, 0.110) | 0.034 |  |
|  | Q3 | 0.118 (0.052, 0.183) | <0.001 |  |
| ALAN _T2_ | Q1 | ref |  | <0.001 |
|  | Q2 | 0.071 (0.015, 0.127) | 0.023 |  |
|  | Q3 | 0.132 (0.068, 0.195) | <0.001 |  |

ALAN: artificial light at night; T1: First Trimester; T2: Second Trimester; Q1-Q3: Categorized into three groups based on percentiles; β: regression coefficient; 95%CI: 95% confidence interval. Model 1: Crude linear regression model; Model 2: Adjusted for age, ethnicity, gravidity, parity, pre-pregnancy body mass index, and conception season; Model 3: Based on model 2, further adjusted for normalized difference vegetation index (NDVI), as well as Principal Component 1 of ambient fine particulate matter (PM_2.5_) and ambient inhalable particulate matter (PM_10_).

Supplementary Table S7, Annual participant enrollment in study: Distribution among all participants, controls, and GDM groups (2013-2020)

| Year | All (N=5720) | Controls (n=4290) | GDM (n=1430) |
| --- | --- | --- | --- |
| 2013 | 482 | 377 | 105 |
| 2014 | 564 | 432 | 132 |
| 2015 | 561 | 440 | 121 |
| 2016 | 902 | 677 | 225 |
| 2017 | 916 | 688 | 228 |
| 2018 | 877 | 658 | 219 |
| 2019 | 1013 | 748 | 265 |
| 2020 | 505 | 370 | 135 |


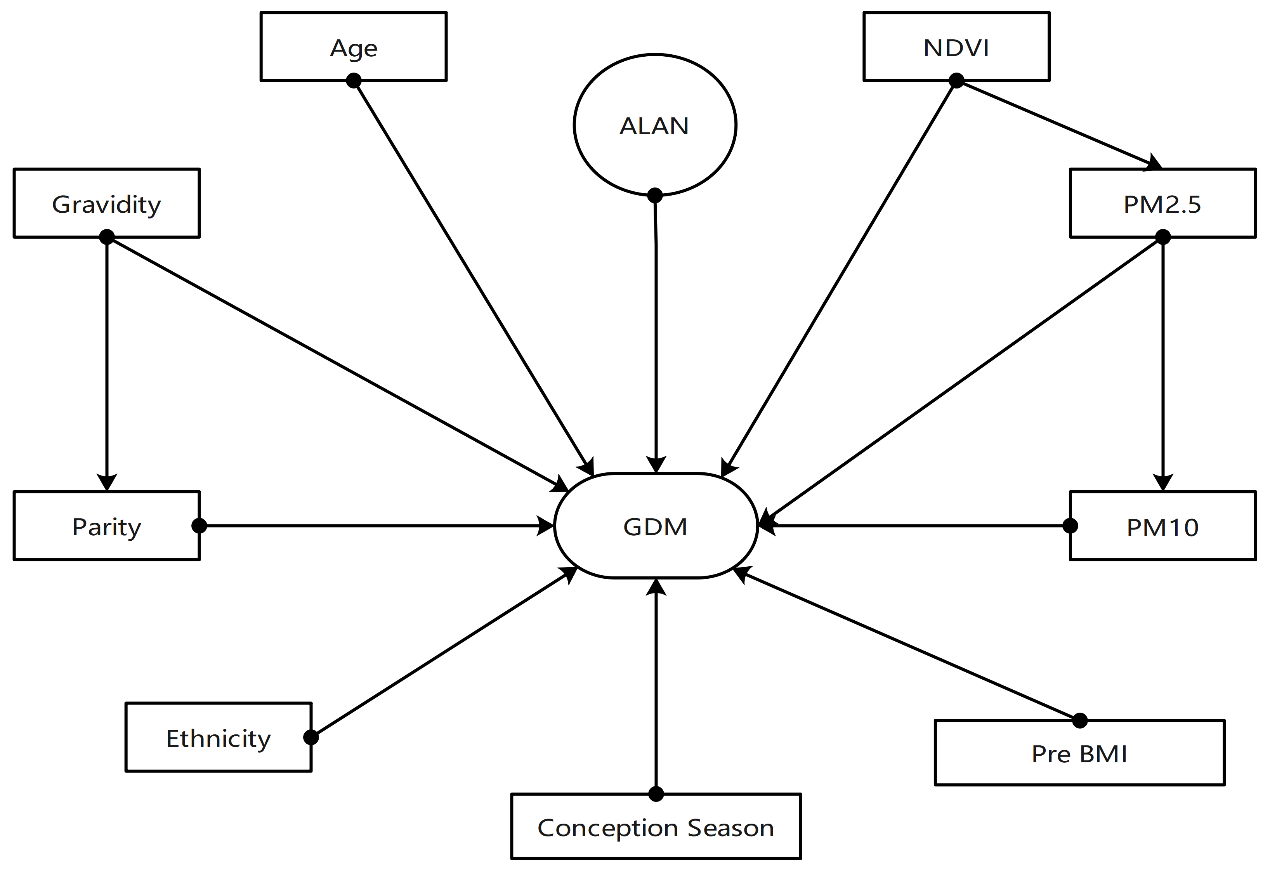


Supplementary Figure S1. Factors influencing GDM: A directed acyclic graph analysis

GDM: Gestational Diabetes Mellitus; ALAN: Artificial Light at Night; NDVI: Normalized Difference Vegetation Index; PM2.5: Fine Particulate Matter; PM10: Inhalable Particulate Matter; Pre BMI: Pre-pregnancy Body Mass Index.
